# Platelets Alter Gene Expression Profile in Human Brain Endothelial Cells in an *In Vitro* Model of Cerebral Malaria

**DOI:** 10.1371/journal.pone.0019651

**Published:** 2011-05-16

**Authors:** Mathieu Barbier, Dorothée Faille, Béatrice Loriod, Julien Textoris, Claire Camus, Denis Puthier, Laurence Flori, Samuel Crocodile Wassmer, Geneviève Victorero, Marie-Christine Alessi, Thierry Fusaï, Catherine Nguyen, Georges E. Grau, Pascal Rihet

**Affiliations:** 1 Laboratoire de Pharmacogenétique des Maladies Parasitaires-EA 864, IFR 48, Faculté de Pharmacie, Aix-Marseille Université, Marseille, France; 2 INSERM U928-TAGC, Aix-Marseille Université, IFR137, Marseille, France; 3 Unité de Recherche en Biologie et en Épidémiologie Parasitaires, UMR 6236-URMITE-IMTSSA, Institut de Recherche Biomédicale des Armées-Antenne Marseille, Marseille, France; 4 INSERM, UMR 626, Faculté de Médecine, Aix-Marseille Université, Marseille, France; 5 Laboratoire de Génétique Animale et Biologie Intégrative, Domaine de Vilvert, INRA AgroParisTech, Jouy-en-Josas, France; 6 Vascular Immunology Unit, Department of Pathology, University of Sydney, Camperdown, Australia; 7 Department of Medical Parasitology, New York University School of Medicine, New York, New York, United States of America; Institut Pasteur, France

## Abstract

Platelet adhesion to the brain microvasculature has been associated with cerebral malaria (CM) in humans, suggesting that platelets play a role in the pathogenesis of this syndrome. *In vitro* co-cultures have shown that platelets can act as a bridge between *Plasmodium falciparum*-infected red blood cells (pRBC) and human brain microvascular endothelial cells (HBEC) and potentiate HBEC apoptosis. Using cDNA microarray technology, we analyzed transcriptional changes of HBEC in response to platelets in the presence or the absence of tumor necrosis factor (TNF) and pRBC, which have been reported to alter gene expression in endothelial cells. Using a rigorous statistical approach with multiple test corrections, we showed a significant effect of platelets on gene expression in HBEC. We also detected a strong effect of TNF, whereas there was no transcriptional change induced specifically by pRBC. Nevertheless, a global ANOVA and a two-way ANOVA suggested that pRBC acted in interaction with platelets and TNF to alter gene expression in HBEC. The expression of selected genes was validated by RT-qPCR. The analysis of gene functional annotation indicated that platelets induce the expression of genes involved in inflammation and apoptosis, such as genes involved in chemokine-, TREM1-, cytokine-, IL10-, TGFβ-, death-receptor-, and apoptosis-signaling. Overall, our results support the hypothesis that platelets play a pathogenic role in CM.

## Introduction

Malaria remains a major problem of public health in tropical developing countries worldwide. Close to 230 million people (World Health Organization) are infected annually with *Plasmodium falciparum*. About 1–2% out of clinical attacks of malaria are complicated by severe manifestations leading to death. The major syndromes of severe malaria are severe anaemia, severe respiratory distress, and cerebral malaria (CM) [1]. Severe malaria predominantly affects children under 5 years of age, non-immune adults, and pregnant women. More generally, the outcome of infection depends on numerous factors, including host and parasite genetics, host age, rates of infection, and host immune status.

CM is a complex neurological syndrome, which accounts for a large proportion of deaths due to malaria. CM is characterized by the sequestration of *P. falciparum*-infected red blood cells (pRBC), leukocytes, and platelets in brain microvessels. It has been proposed that this accumulation of pRBC interferes with microcirculation, and leads to ischemia [2]. In addition, many observations provide evidence that leukocytes and inflammatory cytokines, such as tumor necrosis factor (TNF), are involved in malaria pathogenesis [2], [3]. TNF produced by monocytes activates endothelial cells, thereby increasing the expression of cell adhesion molecules, such as P-selectin, ICAM-1 and VCAM-1. Increased expression of endothelial cell adhesion molecules contributes to enhanced pRBC sequestration, leukocyte and platelets adhesion.

There is a growing body of evidence that platelets (PL) are involved in CM pathogenesis: (i) thrombocytopenia is marked during *P. falciparum* and experimental CM; (ii) PL adhesion to the microvessels has been associated with CM in humans [4], and has been shown to be involved in mouse CM pathogenesis [4], [5]; (iii) *in vitro* co-cultures showed that PL act as a bridge between pRBC and TNF-stimulated endothelium, and contribute to brain endothelial alterations [6], [7].

These studies prompted us to analyze gene expression in endothelial cells co-incubated with PL in an *in vitro* model of CM. Microarray analyses showed that pro-inflammatory cytokines, including TNF, induce distinct gene expression programs in different endothelial cell types [8], [9]; this indicates that the endothelial cell type should be carefully chosen for an *in vitro* model of CM. Some studies have investigated the direct effect of pRBC alone on the human umbilical vein endothelial cell (HUVEC) transcriptome, and revealed changes in the expression of genes involved in tight junction, inflammatory response, and apoptosis [10], [11], [12], whereas co-cultures with human lung microvascular endothelial cells (HLEC) showed that pRBC induces apoptosis through a caspase-dependent pathway [13]. Recently, a genome wide transcriptional profiling of human brain microvascular endothelial cells (HBEC) after exposure with pRBC revealed up-regulated transcripts involved in the immune resfponse, apoptosis, and NF-κB activation cascade [14]. Nonetheless, the influence of PL on endothelial cell gene expression has not been evaluated in those models. We therefore investigated transcriptional changes in HBEC in a tri-partite co-culture model including TNF, pRBC, and PL. This model is a well established *in vitro* model of CM, which has been validated for a number of interactions, as recently reviewed [15]. Here, we report that PL induced transcriptional changes in HBEC related to canonical pathways involved in inflammation or apoptosis.

## Materials and Methods

### Human brain endothelial cells

HBEC-5i were derived by Dorovonis-Zis and colleagues from small fragments of human cortex obtained from patients who died of various causes, devoid of any pathological abnormalities [16]. These cells were immortalized and characterized as described elsewhere [7]. HBEC-5i were grown to confluence in DMEM/F12 medium containing 15 mmol/L HEPES and L-glutamine pH 7.4 (Gibco Life Technologies, Carlsbad USA) supplemented with 10% (v/v) of fetal bovine serum. Conservation of the phenotype of this cell line was checked over passages and cell line was tested negative for mycoplasma contamination every 5 passages.

### Platelets

Venous blood was obtained by venipuncture from one healthy volunteer and anticoagulated with 3.2 g/L buffered sodium citrate. The volunteer had not taken any drugs for at least 1 month. The healthy volunteer gave a written informed consent. Experiments were performed after clearance from human ethics committee of the University of Sydney, which approved the study. Platelet-rich plasma was prepared by centrifugation at 200 g for 15 minutes at room temperature then PL were pelleted by centrifugation of the PL-rich plasma for 6 minutes at 2000 g and washed in HEPES buffer warmed at 37°C (0.137 M NaCl, 2.68 mM KCl, 1 mM MgCl_2_, 1 mM CaCl_2_, 5 mM HEPES, 0.1%glucose, pH = 6.8). PL were then adjusted to 1.10^8^/mL. PL functions were tested by agonist-induced platelet aggregation and PL activation was assessed by flow cytometric measurement of P-selectin in PL-rich plasma and washed PL suspension. PL of the volunteer had a normal reactivity compared to a reference population of 35 healthy controls, and washing steps did not alter either platelet reactivity or activation state.

### Parasites

The IPPAM strain of *P. falciparum* (gift from C. Behr, Institut Pasteur, Paris, France) was obtained by panning on endothelial cells expressing either GPIV (CD36) or ICAM-1 using the method described by Fried and Duffy [17]. Parasites were maintained in continuous culture at 2% hematocrit using type O+ human red blood cells (RBC) as described elsewhere [18]. Uninfected normal red blood cells (NRBC) used as controls were cultured the same way for at least 2 weeks before experiments. Late trophozoite-stage pRBC preparations were enriched to 80 to 85% by gelatin flotation with Plasmion (Fresenius Kabi France, Couvier France) as described elsewhere [19]. NRBC or pRBC were adjusted to 2×10^7^/mL in the co-culture medium composed of RPMI 1640 with the pH adjusted to 6.8 before use.

### Human brain endothelial cells-platelets-parasitized red blood cells co-cultures

HBEC were grown to confluence in 6-well tissue culture plates and were either left unstimulated or incubated overnight with 10 ng/mL recombinant TNF (PeproTech, London UK). HBEC were then washed with PBS and incubated with PL, pRBC or NRBC according to the experimental conditions with the following sequence: HBEC were first incubated with 2×10^8^ PL per well as a first step, washed three times in PBS to remove unbound PL and 4×10^7^ pRBC or NRBC per well were then added as a second step. First and second incubation steps were carried out at 37°C for 90 min. Co-cultures without TNF, PL, pRBC, or NRBC were also performed. Table 1 summarizes all the experimental conditions (n = 24). Each experimental condition was done in triplicate.

**Table 1:** Summary of the 24 experimental conditions^a^.

		-		Platelets (PL)	
		-	TNF (T)	-	TNF (T)
0 h^b^	-	0 h	0h_T	0h_PL	0h_T_PL
	NRBC (NR)	0h_NR	0h_T_NR	0h_NR_PL	0h_T_NR_PL
	pRBC (PR)	0h_PR	0h_T_PR	0h_PR_PL	0h_T_PR_PL
5 h^b^	-	5 h	5h_T	5h_PL	5h_T_PL
	NRBC (NR)	5h_NR	5h_T_NR	5h_NR_PL	5h_T_NR_PL
	pRBC (PR)	5h_PR	5h_T_PR	5h_PR_PL	5h_T_PR_PL

a Labels used on Figure 1. Each experimental condition was done in triplicate.

b Time after co-incubation.

### RNA isolation

After co-incubation, HBEC were washed three times with PBS and either harvested immediately (t = 0 h) or further incubated for 5 h (t = 5 h) and then harvested. Total RNA extraction of HBEC was carried out after harvesting using TRIzol (Gibco Life Technologies, Carlsbad USA) according to the manufacturer's instructions. RNA concentration was determined by reading the absorbance at 260/280 nm. The quality of RNA and the absence of any DNA contamination were checked on a 2100 Bioanalyzer (Agilent Technologies, Santa Clara USA). Each mRNA sample extracted from an individual sample was run on a single microarray.

### cDNA Microarray Hybridization

Hybridizations were carried out for each triplicate of the 24 conditions. All microarray procedures were done at our microarray facilities. Human cDNA microarray were designed and prepared as previously described [20]. PCR amplification was performed as previously described [21], and PCR products were spotted onto nylon membranes (Hybond-N^+^, Amersham, Pharmacia Biotech, UK) with a GMS-427 arrayer (Affymetrix, Santa Clara USA). 9216 clones were spotted; they corresponded to 8780 genes and 436 controls. Production, sensitivity and reproducibility of the microarrays have been previously described [22]. Microarray were hybridized with ^33^P-labelled probes, first with an oligonucleotide sequence common to all spotted PCR products (5′TCACACAGGAAACAGCTATGAC3′) called vector probe to measure the amount of PCR products spotted onto the microarray. Then, after stripping, microarrays were hybridized with complex probes made from 5 µg of retrotranscribed total RNA. Probe preparations, hybridizations and washes were carried out as described previously [22]. Microarrays were exposed on a radioimager during 48 h and scanned using a Fuji BAS5000 machine (Fuji, France). Hybridization signals were quantified using BZscan 2 software [23] locally developed on the TAGC platforms (http://tagc.univ-mrs.fr/welcome/). All data are MIAME compliant and have been loaded into ArrayExpress database (http://www.ebi.ac.uk/microarray-as/ae/). The ArrayExpress accession number of this experiment is E-MEXP-1989.

### Microarray data Analysis

BZscan first applied a criterion of spot quality to select analyzable spots on the microarrays. 4502 genes passed through this selection for all conditions and were analyzed. Since the hybridization signal depends on the amount of PCR products spotted onto the microarray, the signal obtained with the vector probe was used to normalize the signal obtained with complex probes [24]. A specific R library, ‘nylonmagic’ (currently under development, script available upon request), that uses the ‘S4’ system of formal classes and methods was used to process and normalize nylon microarray data [25]. After loading, data obtained with vector probe and complex probes were both corrected for neighborhood effects and local background as described [23]. Quantile normalization was applied to vector probe data (V) and complex probe data (C) to correct for global intensity and dispersion. In a third step, the correction by the vector signal was made by calculating a C/V ratio before log transformation (base 2). The basic approach relies on the assumption that the complex and vector signal intensities are related by a constant factor. However, as reported by others, in many cases, log(C/V) still depends on log(V) [24]. To correct for this bias, we used the method proposed by Talby *et al* [24]. For any sample, C_k_ and V_k_ denote the complex and vector probe signals, and k represents the array element. The curve M_k_ = log(C_k_/V_k_) versus log(V_k_) was fitted using a robust scatter-plot smoother (lowess regression). The normalization adjustment was M′_k_ = M_k_−s(log(V_k_)), where s(log(V_k_)) was the lowess fit to the M versus log(V). In the last step, data were centered relative to the median for each gene.

Unsupervised hierarchical clustering was used to investigate relationships between samples and relationships between genes, and to evaluate the effect of the different sources of variability (TNF, PL, RBC, time-points, and replicates). It was applied to median-centred data, using the Cluster and TreeView programs (average linkage clustering using Euclidian distance as similarity metric).

Statistical analysis was performed using the TIGR MeV (MultiExperiment Viewer) v4.1 software (http://www.tm4.org/mev.html) and the GeneANOVA programs [26]. One-way analysis of variance was applied to identify genes differentially expressed between the treatment groups. Paired t-tests and paired significant microarray analysis method (SAM) were used to evaluate the influence of one particular variability factor (TNF, PL, RBC, and time). The members of each pair differed from each other only with respect to the factor studied. Therefore, the paired design allowed isolating the contribution of the factor of interest. Empirical *P*-values were calculated after 10000 permutations. Moreover, we applied the adaptive false discovery rate (FDR) procedure to control the FDR for the paired t-tests and the paired SAM, respectively [27], [28]. The FDR of a test is the expected proportion of false positive results among the declared significant ones. We generally used an FDR of 5%.

In addition, an ANOVA based on the microarray analysis was used to analyze simultaneously the effect of TNF, PL, RBC, and time on gene expression. A global ANOVA model gives an estimation of the contribution of each factor in the total variation of the whole data set; gene was considered a factor in this model, and interaction terms were included [29]; no multiple test correction was performed. Besides, a local ANOVA allows the determination of each contribution for each gene: it gives an estimation of the variation due to the factor studied, and the significance of the estimate. We applied the adaptive false discovery rate (FDR) procedure to control the FDR for the local ANOVA [27], [28]. We generally used an FDR of 5%. In addition, a two-way ANOVA was performed to analyse for each gene the interaction RBC-by-TNF and the interaction RBC-by-PL. Empirical *P* values were calculated after permutations, and multiple testing was controlled by using the adaptive FDR.

Gene annotation of all 4502 analyzable genes/expressed sequence tags (ESTs) was performed using DAVID database (http://david.abcc.ncifcrf.gov/) [30]. This program, which allows a biological interpretation of gene clusters on the basis of gene ontology (GO) terms and the Kyoto Encyclopaedia of Genes and Genomes (KEGG) pathways (http://www.genome.jp/kegg/), were used to assess whether specific biological pathways were overrepresented among the differentially expressed genes and within specific gene clusters. A score based on Fisher's exact test reflected the probability that the prevalence of a particular term within a cluster was due to chance alone, given the prevalence of that term in the population of all genes under study. The FDR was used to account for multiple testing [27].

The Ingenuity Pathways Analysis (IPA) program permitted the determination of significant networks, top functions and canonical pathways associated with the differentially expressed genes (IPA 5.0, Ingenuity Systems Inc., USA). The IPA program searches the Ingenuity Pathway Knowledge Base (IPKB) for interactions (known from the literature) between the studied gene set and all other genes contained in IPKB and generates a series of networks. The genes selected by the IPA network analysis are called focus genes. Fisher's exact test was used to assign statistical significance, and each network's score was displayed as the −log (*P*-value). Significant networks with a score greater than 3 were selected (*P*<0.001) and top functions associated with these networks are presented. Canonical pathway analysis identified the most significant pathways, from the IPA library of canonical pathways. The association between the input data set and the canonical pathway was scored as the ratio of the number of genes from the data set that map to the pathway divided by the total number of genes that map to the canonical pathway; their significance was assessed by using a *P*-value calculated by using Fisher's exact test.

### Quantitative Real-Time PCR

RNA of HBEC used for Real-Time-quantitative Polymerase Chain Reaction (RT-qPCR) was obtained from another co-culture experiment. cDNAs were prepared from 2 µg of total RNA using random hexamers pd(N)_6_ (GE Healthcare, UK) and M-MLV reverse transcriptase (Invitrogen Life Technologies, Carlsbad USA). RT-qPCR assays were performed on AB-7300 Real-Time PCR system using SYBR PCR Master Mix (Applied Biosystems Life Technologies, Carlsbad USA). Forward and reverse primers used are shown in Table S1. They were designed manually to have primers spanning exon-exon junctions to avoid the amplification of DNA contaminant. PCR were performed using the following conditions: 10 min at 95°C, 15 sec at 95°C and 1 min at 60°C during 40 cycles. Melting curve analysis was performed to check the specificity of the PCR and to verify the amplification efficiency. Relative quantification was calculated with the 2^−ΔΔC(t)^ method: ΔC(t) represents the difference between the threshold cycle of the gene of interest and the threshold cycle of the internal control gene (*ACTB*). Co-cultures were incubated during 5 h, and each tested condition was done twice.

## Results

We used an unsupervised hierarchical clustering method that grouped genes on the vertical axis and samples on the horizontal axis, on the basis of similarity in their expression profiles. The similarities are summarized in a dendogramme in which the pattern and length of the branches reflect the relatedness of the samples. The discrimination between experimental conditions was unsatisfactory on this basis (data not shown). We assumed that the samples were incorrectly classified because of confounding genes, which were not differentially expressed between groups. Therefore, we filtered the genes by using a one-way ANOVA to visualize the effect of experimental conditions. We generated empirical *P*-value for each gene, and selected the genes for which *P*<0.05. We defined 469 informative genes, and we performed unsupervised hierarchical clustering of samples on the basis of the expression of selected genes. Figures 1A and 1B show the classification of samples obtained.

**Figure 1:**
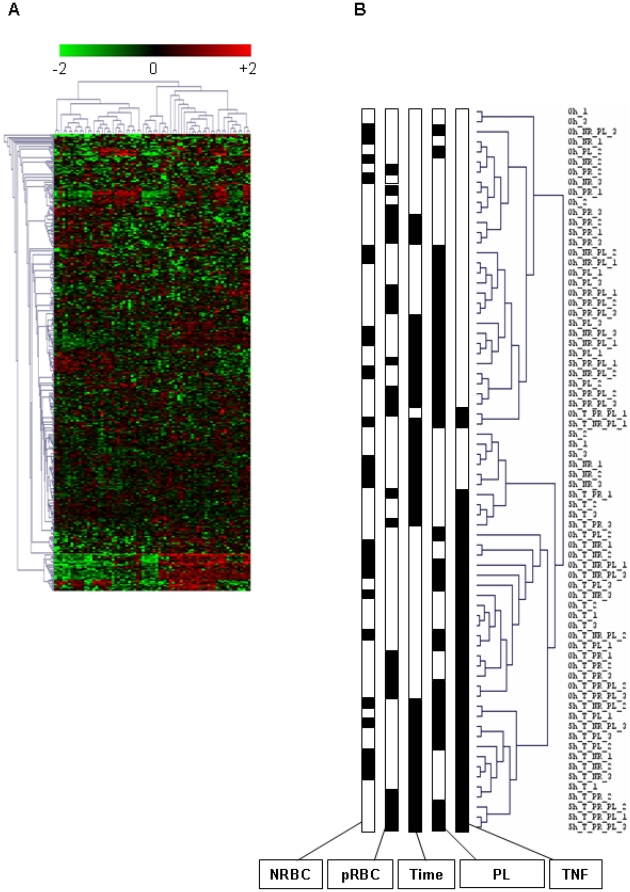
Hierarchical clustering of HBEC responses to NRBC, pRBC, TNF, and platelets. A) Hierarchical clustering of 72 samples was done on the basis of the expression of 469 genes that were selected by using a one-way ANOVA. Each row represents a gene, and each column an experimental sample. Red and green indicate expression levels above and below the median for each gene, respectively. Similarities in gene expression are represented by dendogrammes of samples and genes. B) Dendogramme of samples. 24 experimental conditions and 3 samples per condition are represented. For each condition, the presence (black) or the absence (white) of factors is specified. The factors are NRBC (−/+), pRBC (−/+), co-culture time (0 h and 5 h), PL (−/+), and TNF (−/+).

We further performed a multi-way ANOVA over the whole data set to estimate the contribution of each factor. Such estimates could be performed because we used a fully crossed factorial design, meaning that each state of one factor was found in combination with each state of other factors (Table 1). So, there were 72 measurements for each of the 4502 genes, three per combination of four factors (RBC, TNF, PL and time). Table 2 presents the results of a global ANOVA over the whole data set. It shows that the major source of variation was the gene factor, and that there was a highly significant interaction between the gene factor on the one hand, and time, TNF or PL on the other hand (P<0.0001), whereas the interaction between the gene factor and RBC appeared to be weaker (P = 0.04). In addition, the analysis revealed a significant interaction between gene, PL and TNF (P = 0.00029). Interestingly, RBC was included in high-order interaction terms, namely gene-by-time-by-RBC-by-TNF (P = 0.02), and gene-by-time-by-RBC-by-PL (P = 0.002).

**Table 2:** Multi-way analysis of variance for HBEC gene expression data.

Factor	Sum of square	DF^a^	Mean square	F	*P*-value
gene	849219.05	4501	188.67	274.11	<0.00001
RBC	0.37	2	0.18	0.27	-
TNF	0.17	1	0.17	0.24	-
PL	4.37	1	4.37	6.56	0.01013
time	0.1	1	0.1	0.15	-
gene×RBC	6341.37	9002	0.7	1.02	0.0416
gene×TNF	6497.82	4501	1.44	2.1	<0.00001
gene×PL	4644.08	4501	1.03	1.5	<0.00001
gene×time	5355.23	4501	1.19	1.73	<0.00001
gene×time×TNF	3250.75	4501	0.72	1.09	0.00005
gene×time×PL	3264.17	4501	0.73	1.09	0.00002
gene×TNF×PL	3219.91	4501	0.72	1.07	0.00029
gene×time×RBC×TNF	6177.54	9002	0.69	1.03	0.0214
gene×time×RBC×PL	6256.12	9002	0.69	1.04	0.0026
Residual^b^	176809.89	265625	0.67	-	-
Total	1070570.59	324143	3.3	-	-

a Degree of freedom.

b The residual corresponds to the variance that was not explained by the model.

**Figure 2:**
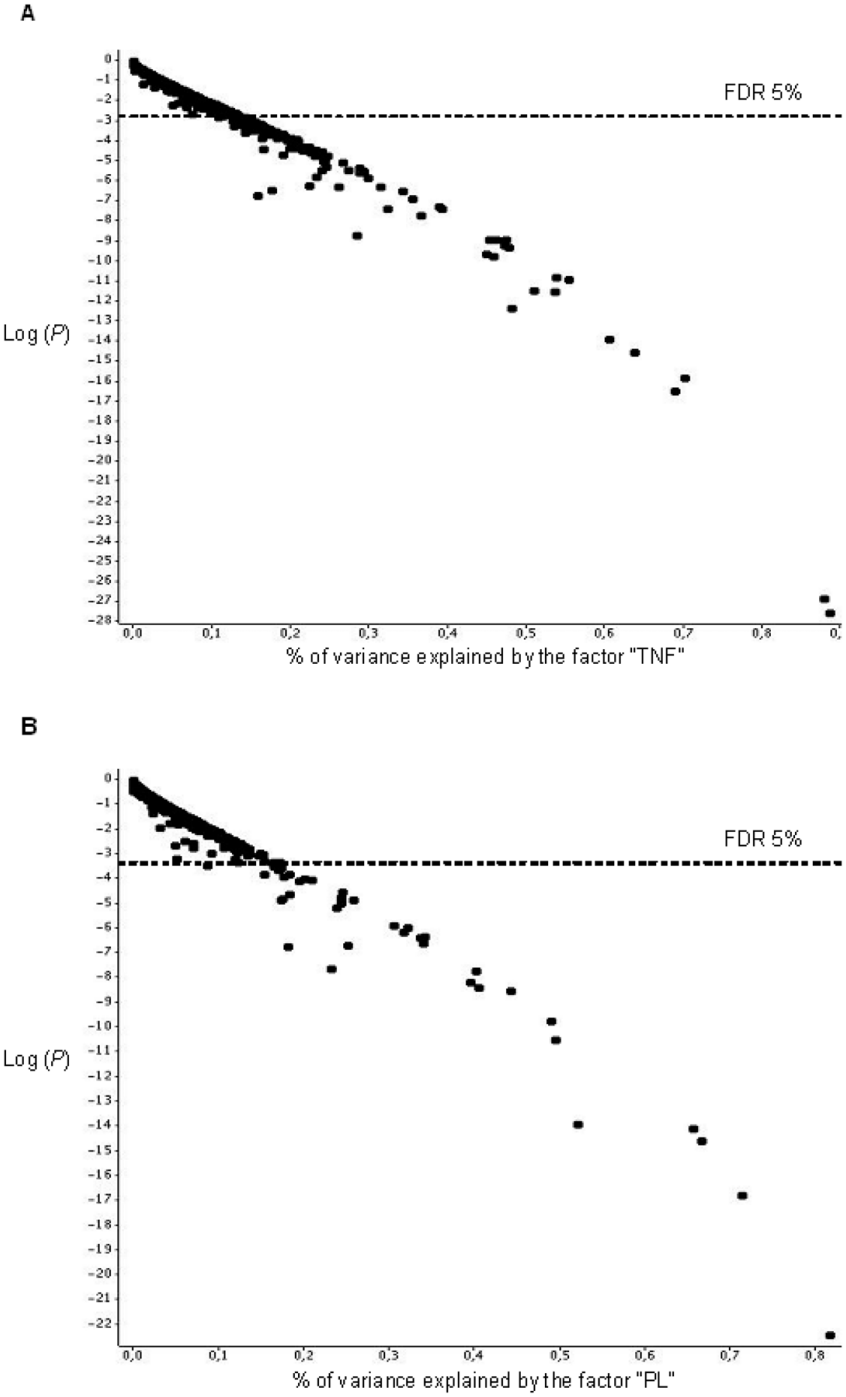
Gene expression variance analysis. Each gene was plotted as a point. The abscissa is the variation due to the factor normalized by the total variation of the gene, and the ordinate is the logarithm of the *P*-value. The nominal *P*-value that corresponded to a FDR of 5% is stated. The results are shown for the TNF (A) and PL (B) factors.

Moreover, we performed an ANOVA for each gene to identify genes showing significant variation in their expression pattern. We assessed the effect of TNF, PL, RBC, and time on gene expression; we did not include interaction terms at this step. Figure 2A and 2B shows a graphical representation of the proportion of gene expression variance due to TNF and PL and the logarithm of *P*-value for each gene. Up to 90% of the variance was explained by TNF or PL for some genes (Figure 2A and 2B), whereas up to 50% of the variance was explained by time (Figure S1A). RBC explained less than 20% of gene expression variance (Figure S1B). Furthermore, we applied a FDR of 5% to account for multiple testing. We identified 39, 143, and 109 genes, the expression of which was significantly altered by PL (Table 3), TNF (Table S2), and time (Table S3), respectively, whereas there was no gene influenced by the factor RBC at this significance level.

**Table 3:** List of genes transcriptionally altered by platelets.

Gene	SAM^a^	t-test	GeneAnova	% variance^b^
*ADAM21*	+	+	+	65,7
*ADAM28*	+	+	+	23,9
*ANGPTL4*	+	+	+	31,8
*BCL3*	+	+	+	24,4
*BIRC2*			+	17,4
*BSCL2*	+	+		
*CCL2*	+	+	+	17,5
*CCL4*	+			
*CCL7*	+			
*CCR7*	+	+	+	19.5
*CD83*	+	(+)		
*CIRH1A*	+		+	17.4
*COPS7B*			+	16.9
*CSEN*	+	(+)	+	17.1
*CTGF*	+	(+)		
*CXCL1*	+	+	+	21
*CXCL3*	+	+	+	49.6
*D2S448*	+	+	+	16.6
*DDIT4*	+		+	18.3
*DNASE1L3*	+	+	+	18.2
*DUSP5*	+		+	8.7
*EHD1*	+	+	+	40.6
*EPHA2*	+	(+)		
*FAM19A4*	+	+	+	24.5
*FCER1G*	+	(+)		
*FOSB*	+	(+)		
*GLB1*	+	+	+	17.4
*H1FX*	+			
*HBEGF*	+	+	+	24.5
*ID3*	+	(+)		
*IER3*	+	+	+	81.7
*IL11*	+	+	+	23.2
*INHBA*	+	+	+	52.2
*INHBB*	+	+	+	33.7
*IRF1*	+	+	+	30.6
*KCNK3*	+	+	+	40.2
*MSC*	+	+	+	39.6
*MUC4*	+	(+)	+	25.9
*NFKBIA*	+	+	+	66.7
*ODC1*	+			
*PAK1IP1*	+	(+)	+	15.4
*PDE1A*	+	+	+	34.2
*PDE7B*	+			
*PGCP*	+	+	+	18.3
*PLK2*	+	+	+	34
*PSFL*			+	17.5
*PTK6*	+	+	+	32.3
*RAVER1*	+	(+)		
*SACS*	+	+		
*SLAMF8*	+	+	+	71.4
*SORL1*	+	(+)	+	20.2
*STK23*	+	+	+	44.3
*TCEAL8*				
*TNNI3*	+	+		
*TPD52L1*	+	+	+	17.8
*TRAF4*	+			
*UAP1*	+			
*UCK2*	+			

+: informative genes with a FDR 5%.

(+): informative genes with a t-test 5% FDR 10%.

a Significance Analysis of Microarray.

b percentage of variance due to PL from multi-way ANOVA analysis.

**Figure 3:**
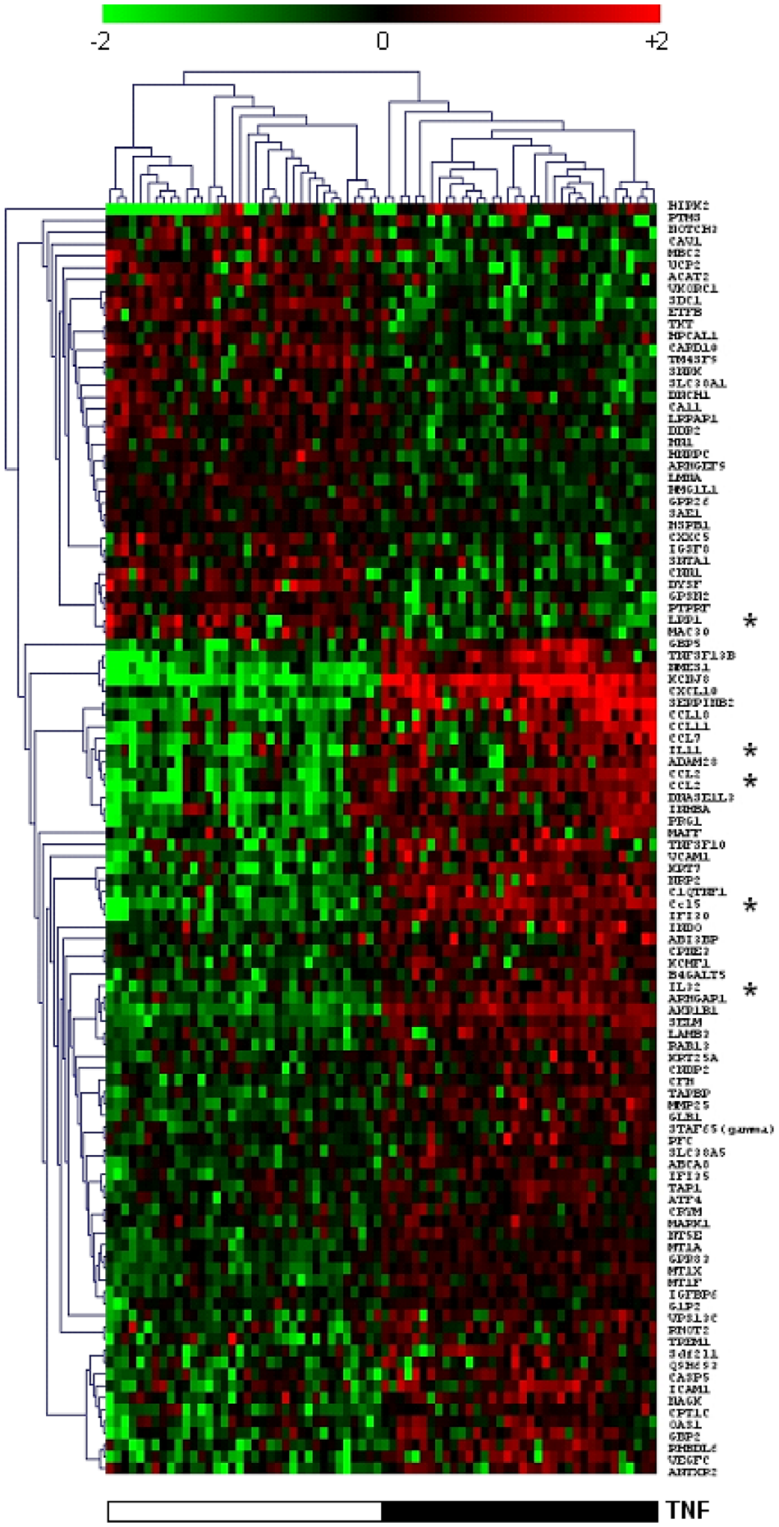
Hierarchical clustering of TNF-induced genes. Hundred and seven TNF-induced genes were selected on the basis of a Welch t-test and a FDR of 5%. The samples obtained from HBEC stimulated with TNF were compared with those from HBEC incubated without TNF. The presence (black) and the absence (white) of TNF are shown. Genes marked with an asterisk were validated by RT-qPCR.

**Figure 4:**
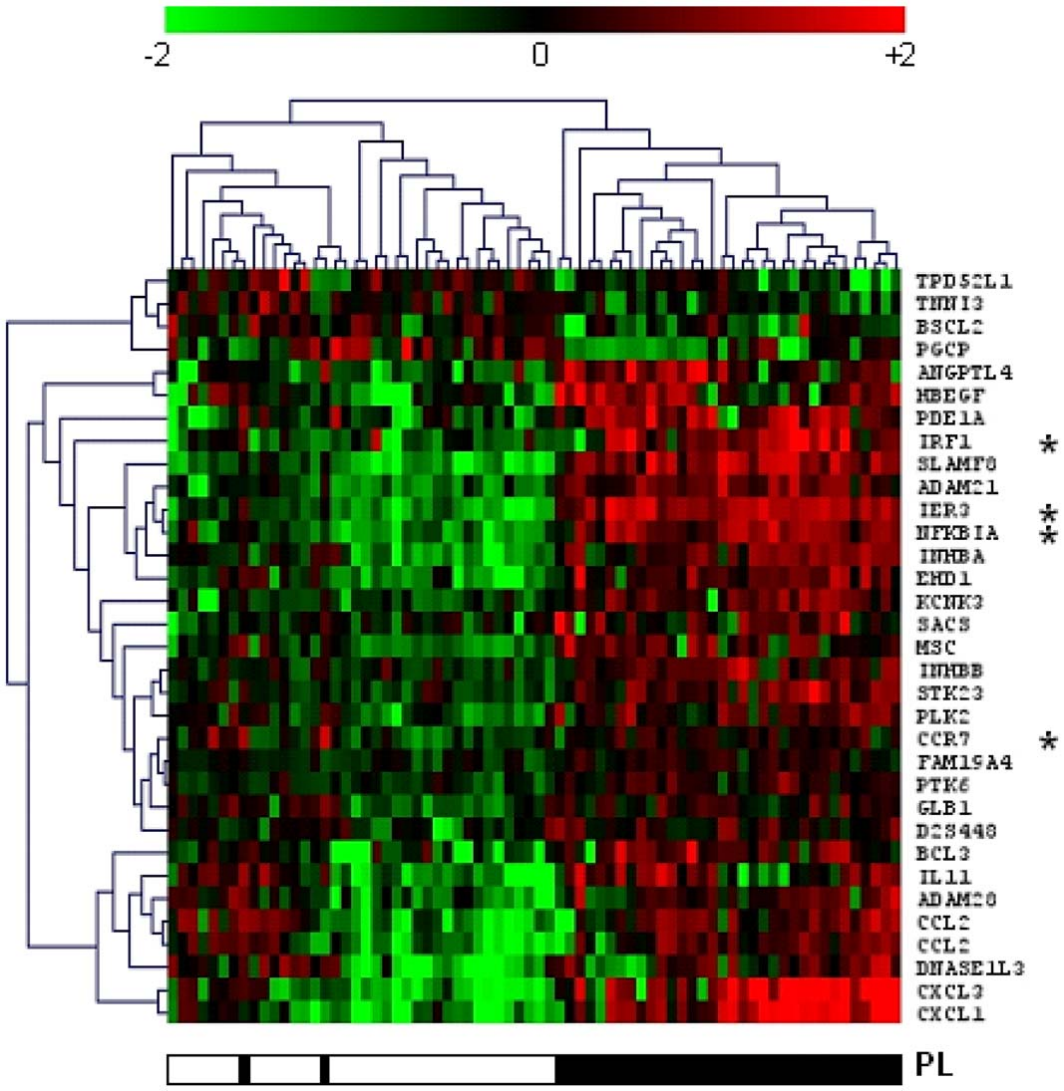
Hierarchical clustering of platelet-induced genes. A Welch t-test and a FDR of 5% led to identify 32 PL-induced genes. The samples obtained from HBEC co-cultured with PL were compared with those from HBEC incubated without PL. The presence (black) and the absence (white) of PL are shown. Genes marked with an asterisk were validated by RT-qPCR.

We applied additional statistical analyses to identify genes, the expression of which was altered by one particular factor. We used the paired t-test and paired SAM method on the full data set (n = 4502), and applied a FDR of 5%. Paired t-tests yielded lists of 107 and 32 genes that showed a differential expression between experimental conditions with and without TNF and PL, respectively. Hierarchical clusterings of TNF- and PL-informative genes yielded with the t-test are presented in Figures 3 and 4 respectively. Seventy-one genes were also differentially expressed when comparing gene expression at 0 h and 5 h. Table 3 shows the genes transcriptionally modified by the platelets (PL) as determined by the three different methods. Comparison of the methods of analysis indicates that SAM identified the largest number of differentially expressed genes (54 genes), whereas ANOVA and paired t-tests identified 39 and 32 genes respectively. When gene lists obtained with the t test were compared to those obtained with other methods, t-test exhibited 91% agreement (29 out of 32) with ANOVA, and 100% agreement with SAM (32 out of 32). Similar results were obtained when the genes transcriptionally modified by TNF and time were determined by the different methods (supplementary Tables S2 and S3, respectively). Overall, the different methods confirmed that the expression of a significant number of genes was influenced by TNF, platelets or time.

In contrast, there was no gene showing differential expression when comparing experimental conditions with NRBC and those with pRBC with a FDR of 5%. It should be stressed, however, that the SAM method identified 11 genes when we applied a FDR of 35%. Interestingly, *TARBP2*, *ALS2CL*, *P4HA1*, *KLK7*, *POR*, *RANBP9*, *Q9H693*, and *SCARB1* that were identified by SAM were also differentially expressed as determined by ANOVA with a nominal threshold of *P*<0.01. Besides, we assumed that the effect of pRBC *per se* was sub-optimal, and that it could be detectable when accounting for putative interaction with TNF and/or PL. We found evidence of an interaction between gene, pRBC, TNF and time (*P* = 0.02) and between gene, pRBC, PL and time (*P* = 0.002) by using the global GeneANOVA (Table 2). This indicates that the effect of the interactions between pRBC and TNF and between pRBC and PL depended on time. Therefore, we separately analyzed gene expression at time 0 h and 5 h, performed a two-way ANOVA, and applied a FDR of 5% to identify genes involved. There was a significant interaction between pRBC and TNF at time 5 h for 4 genes (*KTN1*, *RRBP1*, *GLIPR1*, *CORO2B*) and a significant interaction between pRBC and PL for 4 genes at time 0 h (*ADAMTS16*, *RREB1*, *EFNB3*, *PLXDC1*), and for 3 genes at time 5 h (*ASB12*, *AP3M2*, *LAMP1*). This suggests that pRBC act in interaction with PL and TNF to induce a transcriptional response in HBEC.

The Gene Ontology (GO) terms and KEGG annotations of genes included in the different lists were analyzed by using DAVID tools. Table 4 shows the results of the functional annotation analysis. There was an over-representation of GO terms, such as “immune response”, “cell adhesion” and “apoptosis” in the list of genes regulated by TNF. The analysis of GO terms of genes regulated by PL showed an over-representation of “apoptosis” and “cytokine activity”. The analysis of GO terms of the 469 genes identified by the one-way ANOVA yielded similar results (data not shown).

**Table 4:** Functional annotation clustering of genes differentially expressed in the presence of TNF, platelets, and between 0 h and 5 h of incubation.

	Functional annotation (GO terms)	% of genes	*P*-value
TNF-regulated genes^a^:164 genes annotated/185 genes	immune response	16	6×10^−6^ b
	plasma membrane	29	9×10^−4^ b
	localization	29	7×10^−3^
	cell adhesion	10	3×10^−2^
	regulation of signal transduction	8	4×10^−2^
	apoptosis	11	5×10^−2^
	protein kinase cascade	7	5×10^−2^
PL-regulated genes^a^:53 genes annotated/58 genes	cytokine activity	15	2×10^−5^ b
	apoptosis	25	2×10^−4^ b
	I-kappaB kinase/NF-kappaB cascade	8	2×10^−2^
	B-cell activation	6	3×10^−2^
	heparin binding	6	3×10^−2^
Time-regulated genes^a^:136 genes annotated/158 genes	protein kinase cascade	10	1×10^−3^ b
	response to biotic stimulus	6	2×10^−2^
	intracellular	74	3×10^−2^
	enzyme linked receptor protein signaling	6	3×10^−2^
	cell maturation	3	3×10^−2^
	JNK cascade	3	4×10^−2^

a Informative genes identified by either paired t-test, paired SAM or ANOVA analyses.

b FDR<5%.

**Figure 5:**
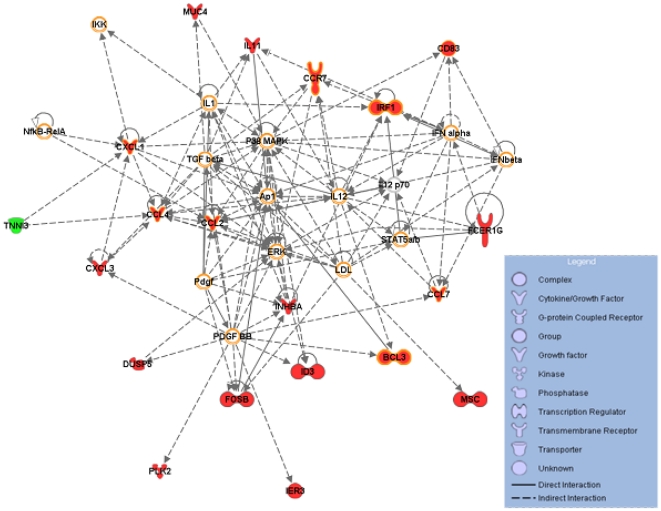
A gene network of platelet-regulated genes. This network is the most significative network among five significative networks obtained using the 58 PL-regulated genes with IPA (supplementary data). Twenty genes of this network are differentially expressed in HBEC in presence of PL (colored in green for the down-regulated gene and red for up-regulated genes). The molecules hedged in orange belong to the main canonical pathways linked to this network (Acute phase response-, B cell receptor-, Chemokine-, Erythropoietin-, Glucocorticoid receptor-, IL6-, IL8-, IL10-, IL12-, PPAR-, RAR-, TREM-1-signaling, IRF, LXR/RXR, PPARa/RXRa, RAR activation, Role of pattern recognition receptor, Role of PKR in IFN induction, Hepatic cholestasis, Hepatic fibrosis).

We used IPA software to visualize differentially expressed genes in networks of interacting genes and to identify the biological functions that were the most significant in our data set. We observed that 56 of the 58 genes differentially expressed between experimental conditions with and without PL could be assigned to one among five related networks at the 0.001 significance level (Table S4). The analysis of the canonical pathways indicated that HBEC has altered activities of several functional pathways, including cytokine-, chemokine-, death receptor-, apoptosis-, erythropoietin-, and TGFβ- signaling (Table S5). Figure 5 shows the most significant network, the top functions of which were haematological system development and immune system. We identified, in this network, several canonical pathways significantly represented; these included several cytokine- and chemokine- signaling pathways, but also glucocorticoid receptor-, PPAR- and TREM1-signaling pathways.

**Figure 6:**
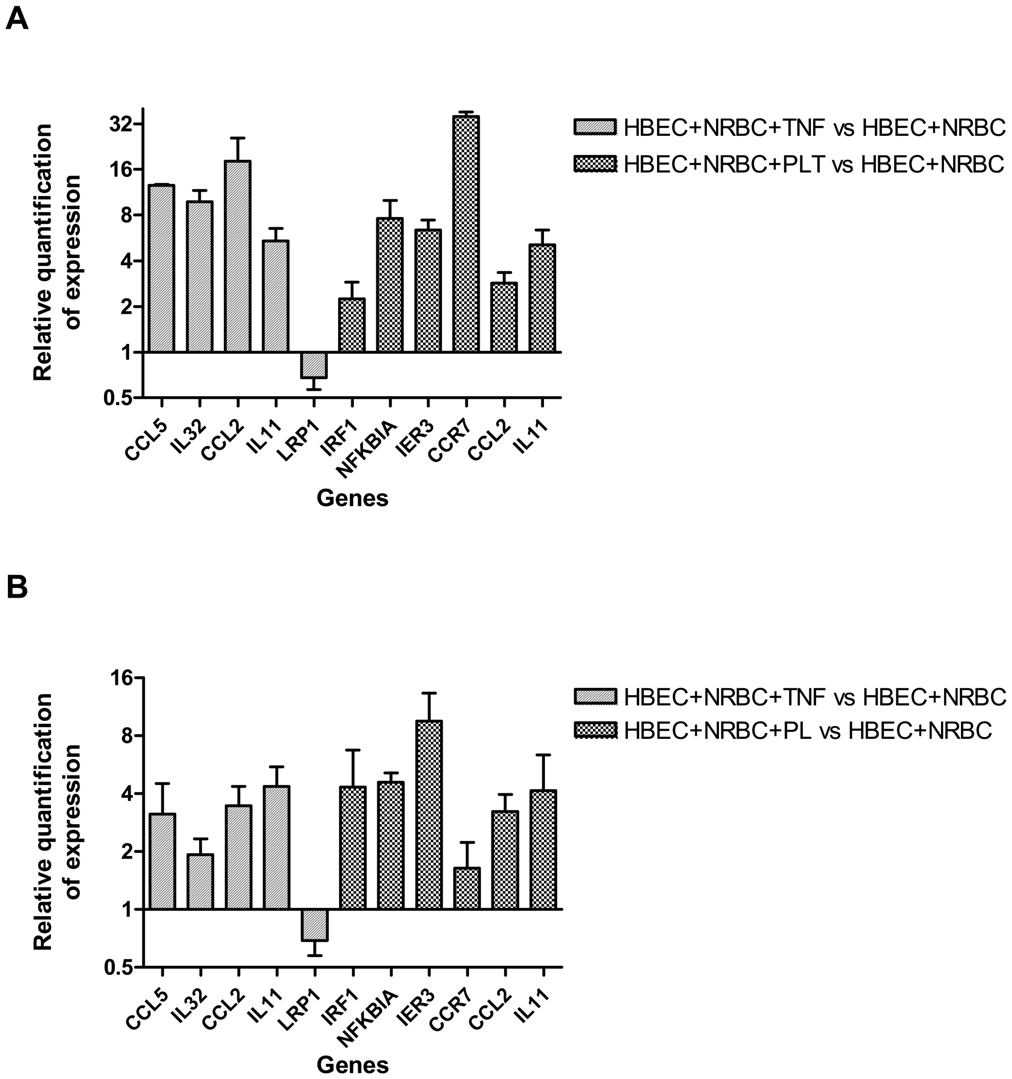
Comparison of the expression of selected genes assessed by RT-qPCR and by microarray technology. Five TNF-induced genes and 6 PL-induced genes were selected from the list of genes identified through Welch t-tests and multiple test corrections. For 2 genes (*CCL2* and *IL11*), gene expression was regulated by TNF and PL. HBEC were co-cultured with NRBC in the presence or the absence of either TNF or PL to measure the expression of TNF- or PL-specific genes, respectively. A) Gene expression was assessed by RT-qPCR. Data were analyzed by the 2^−ΔΔC(t)^ method with *ACTB* as a control gene. The n-fold value represents the mean of TNF- and PL-induced expression levels compared with control conditions. Error bar represents SD between duplicates. B) Gene expression was assessed by microarray technology. The n-fold value was calculated on the basis of normalized data when comparing the levels of TNF- and PL-induced expression with those of control conditions. Error bar represents SD between triplicates.

We further analyzed the expression of 11 selected genes by RT-qPCR to confirm their gene expression pattern. Transcript levels for each of the 11 genes were quantified by using SYBR Green technology. Figure 6A shows differences in their expression i) between endothelial cells incubated with TNF and those incubated without TNF and ii) between endothelial cells cocultured with PL and those incubated without PL. Microarray and RT-qPCR results were consistent, except for *CCR7*. Most importantly, the positive or negative changes of expression remained consistent. As expected, there was a difference of sensitivity between microarray and RT-qPCR methods. RT-qPCR results of selected genes ranged from 1.5-fold to 36-fold regulation (Figure 6A), whereas microarray results ranged from 1.5-fold to 10-fold regulation for the same genes (Figure 6B). The fold changes in transcript abundance of *CCR7* strongly differed between RT-qPCR and microarray techniques. Transcript levels for *CCR7* were the lowest ones among those for the 10 selected genes by using RT-qPCR, and very low signal intensities were measured for *CCR7* by using the microarray technology; this indicates that differences in gene expression are more difficult to quantify by using microarray technology for low transcript levels.

## Discussion

Here we have investigated the respective effects of TNF, PL and pRBC on human brain endothelial cells by profiling gene expression from *in vitro* co-cultures. Unsupervised hierarchical clustering of samples indicated an effect of a short exposure to PL, besides a strong effect of a prolonged exposure to TNF. We applied stringent statistical analyses to identify genes differentially expressed between the experimental conditions, and we searched for over-representation of functional annotations to identify pathways involved.

As expected, TNF regulated the expression of genes involved in inflammation, such as *IL32*, *IL11*, *CCL18*, *CXCL10*, *CCL2*, and *CCL5*, and apoptosis, such as *SERPINB2*, *TNFSF13B* and *TNFSF10*. Similar results have been previously obtained with non-cerebral endothelial cells [8], [31], [32], [33]. Overall, our results confirm that endothelial cells activated by TNF contribute to endothelial cell apoptosis and leukocyte chemotaxis and activation. Interestingly, we detected a significant down-regulation of *LRPAP1* and *LRP1* in TNF-stimulated HBEC. LRP1 that binds to LRPAP1 has been shown to be involved in the clearance of the β-amyloid protein [34], which accumulates in the brain of patients with Alzheimer's disease [35]. Since the β-amyloid protein has been also detected in brains during human and mouse CM [36], [37], our results suggest that TNF inhibits the capacity of HBEC to eliminate the β-amyloid protein from brain. This further supports the hypothesis that CM and Alzheimer' disease share some common mechanisms of pathogenesis [37].

Here we show that platelets dramatically modulate the expression of genes involved in inflammation and apoptosis. The analysis of canonical pathways revealed the effect of PL on cytokine-, chemokine-, TGFβ-, death-receptor-, apoptosis-, erythropoietin-, and TREM1-signaling. These observations are in line with evidence that i) TGFβ participates in HBEC apoptosis induced by PL [38], ii) erythropoietin promotes endothelial cell integrity and angiogenesis [39] and has been associated with resistance against CM in humans and mice [40], [41], iii) TREM-1, which is expressed on myeloid and endothelial cells, has been shown to be involved in the innate inflammatory response and sepsis [42], [43], and its ligand is expressed on human PL [44]. All together, these data support the hypothesis that PL induce HBEC gene expression changes affecting the outcome of malaria infection. It should be stressed that we used platelets from one single donor, and that this is a limitation in our approach. However, those platelets did not show any abnormalities in term of reactivity and probably induced a typical response of HBEC.

It is likely that both TNF and PL can contribute to apoptosis and inflammation, suggesting that TNF and platelets work in synergy, contributing to the same cell response. Indeed the expression of some genes, including *ADAM28*, *DNASEIL3*, and *CCL2*, appeared to be regulated both by TNF and platelets, and the expression of other genes (*GPR83*, *IER3*, *RAB4*, and *HLA-DRB1*) was altered by an interaction between TNF and PL. This interaction underlines the role of TNF in the induction of PL adherence to cerebrovascular endothelium, notably *via* the overexpression of ICAM-1 on EC surface [45] and confirms that PL potentiate the pro-apoptotic effect of TNF on HBEC [7]. Interestingly, TNF and PL also induced the expression of genes with an anti-inflammatory and/or anti-apoptotic activity, such as *IL11* and *INHBA*. It should be stressed that we analyzed gene expression immediately and 5 hours after co-cultures, whereas apoptosis was detected in endothelial cells only 48 h after platelets co-incubation [7]. Chakravorty *et al* recently proposed that the balance between positive and negative regulation will determine endothelial pathology during malaria infection [12]. Our result highlights that a pro-apoptotic stimulus also induces regulatory genes, and fits well with the observation of a transient dysfunction of the blood brain barrier in human severe malaria [46].

When compared to previous reports [10], [12], [13], [14], we found that the likelihood of pRBC-induced transcriptional changes were much lower than those triggered by PL or TNF, i.e. required a FDR of 35% instead of 5%. Such discrepancies may reflect differences in endothelial cell lines, in the durations of exposure to pRBC, and in parasite strains. For instance, we co-cultured HBEC and *P. falciparum* from the IPPAM strain with a polymorphic adhesion phenotype, equally CD36- and ICAM-1-binding, whereas others incubated endothelial cells (HUVEC, HLEC, HBEC…) with *P. falciparum* ItG and ITO4-A4 (ICAM-1 binding) or 3D7 and FCR3 (CD36-binding) strains. In addition, we co-cultured HBEC and pRBC for a short time to detect early events, whereas others detected transcriptional changes after a prolonged co-culture, which may favour the effect of pRBC on endothelial cells [10], [12], [13], [14]. We assume that our system was not optimal to detect the effect of pRBC *per se*, but might be more relevant physiologically to analyse the early effect of adherent pRBC. Besides, we cannot exclude that we missed some genes of interest that were not spotted on the microarrays. Interestingly, Viebig *et al.*, Pino *et al.*, Tripathi *et al.*, and Chakravorty *et al.* collectively provided evidence that pRBC represent a pro-inflammatory (cell adhesion molecules, chemotaxis and chemokine) and a pro-apoptotic stimulus for endothelial cells [10], [12], [13], [14]. This suggests that pRBC together with TNF and PL contribute to similar endothelial responses. Using a global ANOVA, we observed a time-dependent interaction between pRBC and TNF and between pRBC and PL, and we further identified some genes by using a two-way ANOVA, whose expression was influenced by these high-order interaction terms. Although the power may be insufficient to identify such genes in our analysis, our results suggest that pRBC together with TNF or PL contribute to endothelial responses.

In conclusion, this study shows that both TNF and PL dramatically alter gene expression levels in HBEC, and suggests that pRBC act in interaction with TNF and PL to induce transcriptional changes in HBEC. It also indicates that TNF and PL contribute to HBEC apoptosis. This study further supports the pathogenic role of platelets, and points out several altered canonical pathways, such as chemokine-, TGFβ-, death-receptor, erythropoietin-, and TREM1-signaling. We speculate that these observations can be used to develop novel strategies to prevent CM.

## Supporting Information

Figure S1 **Gene expression variance analysis for time (A) and RBC (B) factors.** Each gene was plotted as a point. The abscissa is the variation due to the factor normalized by the total variation of the gene, and the ordinate is the logarithm of the P-value.
(DOC)

Table S1 Primers used for the RT-qPCR.
(DOC)

Table S2 List of genes transcriptionally altered by TNF.
(DOC)

Table S3 List of genes transcriptionally altered by time.
(DOC)

Table S4 IPA Network analysis based on the 58 platelet-regulated genes.
(DOC)

Table S5 IPA Canonical Pathways analysis based on the 58 platelet-regulated genes.
(DOC)
