# Supplementary material for: Platelets Alter Gene Expression Profile in Human Brain Endothelial Cells in an In Vitro Model of Cerebral Malaria
Source: PLoS One. 2011 May 16;6(5):e19651. doi: 10.1371/journal.pone.0019651 (PMC3095604; doi:10.1371/journal.pone.0019651)
Supplement: Figure S1 — Gene expression variance analysis for time (A) and RBC (B) factors. Each gene was plotted as a point. The abscissa is the variation due to the factor normalized by the total variation of the gene, and the ordinate is the logarithm of the P-value. (DOC) [file pone.0019651.s001.doc]

**Figure S1. Gene expression variance analysis for time (A) and RBC (B) factors.**

**A**


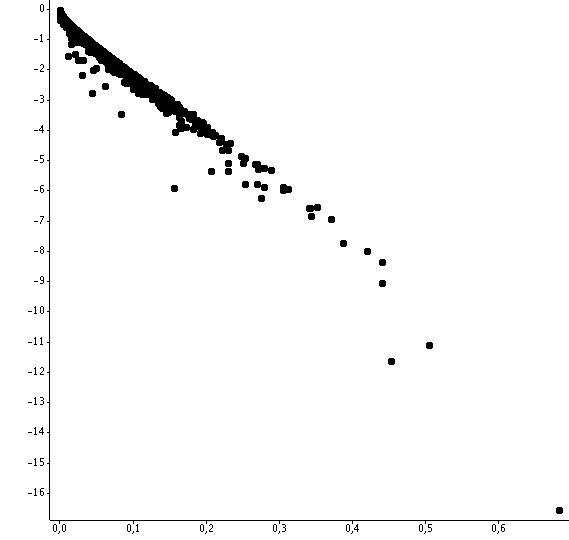


Log (P)

FDR 5%

% of variance explained by the factor “Time”

**B**

**
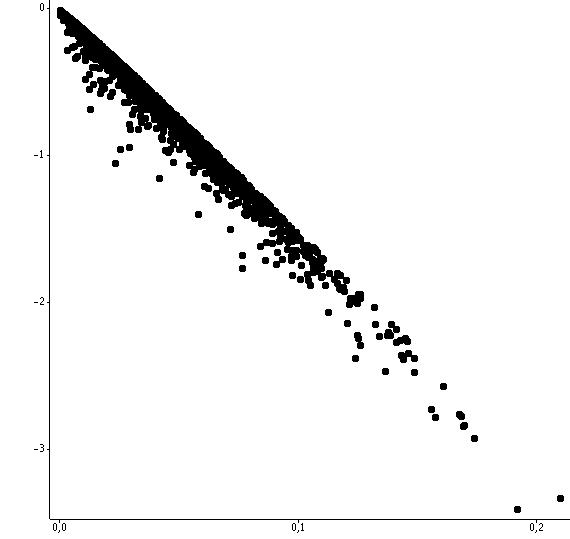
**

Log (P)

% of variance explained by the factor “RBC”
