# Supplementary material for: Platelets Alter Gene Expression Profile in Human Brain Endothelial Cells in an In Vitro Model of Cerebral Malaria
Source: PLoS One. 2011 May 16;6(5):e19651. doi: 10.1371/journal.pone.0019651 (PMC3095604; doi:10.1371/journal.pone.0019651)
Supplement: Table S1 — Primers used for the RT-qPCR. (DOC) [file pone.0019651.s002.doc]

**Table S1. Primers used for the RT-qPCR.**

|  | Primers 5' to 3' | |
| --- | --- | --- |
| Gene | Forward | Reverse |
| *ACTB* | CCAACCGCGAGAAGATGA | CCAGAGGCGTACAGGGATAG |
| *CCL2* | TCGCTCAGCCAGATGCAAT | CCACAATGGTCTTGAAGATCAC |
| *CCL5* | ATCTGCCTCCCCATATTCC | TTTCGGGTGACAAAGACGAC |
| *CCR7* | TCATGGACCTGGGGAAAC | TTGACACAGGCATACCTGG |
| *IER3* | TTCTCTACCCTCGAGTGGTC | CACACCCTCTTCAGCCATC |
| *IL11* | GACATGAACTGTGTTTGCCG | GAATTTGTCCCTCAGCTGTG |
| *IL32* | GAAGGTCCTCTCTGATGACA | AAGTAGAGGAGTGAGCTCTG |
| *IRF1* | CATTCACACAGGCCGATAC | CCACATGACTTCCTCTTGG |
| *LRP1* | TATCGACGCCCCTAAGAC | GCCTTACTCTGTGGACAAATC |
| *NFKBIA* | AGAACAACCTGCAGCAGACT | TAGACACGTGTGGCCATTG |
