# Supplementary material for: Platelets Alter Gene Expression Profile in Human Brain Endothelial Cells in an In Vitro Model of Cerebral Malaria
Source: PLoS One. 2011 May 16;6(5):e19651. doi: 10.1371/journal.pone.0019651 (PMC3095604; doi:10.1371/journal.pone.0019651)
Supplement: Table S2 — List of genes transcriptionally altered by TNF. (DOC) [file pone.0019651.s003.doc]

**Table S2. List of genes transcriptionally altered by TNF.**

| Gene | SAM | t-test | GeneAnova |
| --- | --- | --- | --- |
| *ABCA8* | + | + | + |
| *ABI3BP* | + | + |  |
| *ACAT2* | + | + | + |
| *ADAM28* | + | + | + |
| *AKR1B1* | + | + | + |
| *ANTXR2* | + | + | + |
| *APOL3* | + |  | + |
| *ARHGAP1* | + | + | + |
| *ARHGEF9* |  | + | + |
| *ATF4* | + | + | + |
| *ATP6V0E* | + |  |  |
| *B4GALT5* | + | + | + |
| *BAG5* | + |  |  |
| *BLVRA* | + |  |  |
| *C1QTNF1* | + | + | + |
| *CA11* | + | + | + |
| *CACNA2D2* | + |  |  |
| *CALB2* | + |  | + |
| *CARD10* | + | + | + |
| *CASP5* | + | + | + |
| *CAV1* | + | + | + |
| *CCL11* | + | + | + |
| *CCL18* | + | + | + |
| *CCL2* | + | + | + |
| *Ccl5* | + | + | + |
| *CCL7* | + | + | + |
| *CCR7* | + |  |  |
| *CD44* | + |  |  |
| *CD74* | + |  | + |
| *CDC20* |  |  | + |
| *CDC42BPA* | + |  | + |
| *Cdsn* | + |  |  |
| *CFH* | + | + | + |
| *CLDN1* | + |  |  |
| *CHKA* |  |  | + |
| *CNDP2* | + | + | + |
| *CNN1* | + | + | + |
| *CPNE3* | + | + | + |
| *CPT1C* | + | + | + |
| *CRYM* | + | + | + |
| *CUL3* | + |  |  |
| *CXCL10* | + | + | + |
| *CXXC5* | + | + | + |
| *CYP2S1* | + |  |  |
| *DATF1* | + |  | + |
| *DDR2* | + | + | + |
| *DGCR6L* | + |  | + |
| *DNAJB9* | + |  | + |
| *DNASE1L3* | + | + | + |
| *DNCH1* | + | + | + |
| *DYSF* | + | + | + |
| *EDA2R* | + |  |  |
| *EHD1* | + |  |  |
| *ENO2* | + |  |  |
| *ETFB* | + | + | + |
| *F8A1* | + |  |  |
| *FGF4* | + |  |  |
| *FPR1* | + |  |  |
| *FTMT* | + |  |  |
| *G1P2* | + | + | + |
| *GBP2* | + | + | + |
| *GBP5* | + | + | + |
| *GLB1* | + | + | + |
| *GPA33* | + |  |  |
| *GPR26* |  | + |  |
| *GPR83* | + | + | + |
| *GPSN2* | + | + | + |
| *GRPEL1* | + |  |  |
| *HIPK2* | + | + | + |
| *HIST1H3H* | + |  | + |
| *HMG1L1* |  | + | + |
| *HN1* | + | + | + |
| *HNRPC* | + | + | + |
| *HPCAL1* | + | + | + |
| *HSF1* | + |  | + |
| *HSF2* | + |  |  |
| *HSPB1* | + | + | + |
| *IBRDC3* | + |  | + |
| *ICAM1* | + | + | + |
| *IFI30* | + | + | + |
| *IFI35* | + | + | + |
| *IGFBP6* | + | + | + |
| *IGSF8* | + | + | + |
| *IL11* | + | + | + |
| *IL13* | + |  |  |
| *IL32* | + | + | + |
| *IL8* |  |  | + |
| *INDO* | + | + | + |
| *INHBA* | + | + | + |
| *KCMF1* | + | + | + |
| *KCNJ8* | + | + | + |
| *KCNK6* | + |  |  |
| *KCNMA1* | + |  |  |
| *KRT25A* | + | + | + |
| *KRT7* | + | + | + |
| *LAMB3* | + | + | + |
| *LAP3* | + |  | + |
| *LGI4* | + |  | + |
| *LGMN* | + |  |  |
| *LMNA* | + | + | + |
| *LRP1* | + | + | + |
| *LRPAP1* | + | + | + |
| *LRRC7* | + |  |  |
| *MAC30* | + | + | + |
| *MAFF* | + | + | + |
| *MARK1* | + | + | + |
| *MBC2* | + | + | + |
| *MFNG* | + |  | + |
| *MMP25* | + | + | + |
| *MT1A* | + | + | + |
| *MT1F* | + | + | + |
| *MT1X* | + | + | + |
| *MYNN* | + |  |  |
| *NAGK* | + | + | + |
| *NEBL* | + |  |  |
| *NDUFA11* |  |  | + |
| *NDUFC1* |  |  | + |
| *NFKB2* | + |  | + |
| *NMES1* | + | + | + |
| *NOTCH3* | + | + | + |
| *NRP2* | + | + | + |
| *NT5E* | + | + | + |
| *OAS1* | + | + | + |
| *OTOF* | + |  |  |
| *PAFAH1B3* | + |  | + |
| *PCDHB6* |  |  | + |
| *PDZRN3* | + |  | + |
| *PFC* | + | + | + |
| *PICALM* | + |  | + |
| *PPARG* | + |  |  |
| *PPP3CC* | + |  |  |
| *PRG1* | + | + | + |
| *PTMS* | + | + | + |
| *PTPLA* |  |  | + |
| *PTPRF* | + | + | + |
| *Q9H693* | + | + | + |
| *RAB13* | + | + | + |
| *RAB5A* | + |  |  |
| *RASGEF1C* | + |  |  |
| *RGS10* | + |  |  |
| *REXO2* |  |  | + |
| *RHBDL6* | + | + | + |
| *RHOT1* | + |  | + |
| *RHOT2* | + | + |  |
| *RNF5* | + |  |  |
| *SAE1* |  | + | + |
| *SDC1* | + | + | + |
| *Sdf2l1* | + | + | + |
| *SELM* | + | + | + |
| *SERPINB2* | + | + | + |
| *SLC2A5* | + |  |  |
| *SKP1A* |  |  | + |
| *Slc2a6* | + |  | + |
| *SLC38A1* | + | + | + |
| *SLC38A5* | + | + | + |
| *SLC9A9* | + |  | + |
| *SMARCC2* | + |  | + |
| *SNRK* | + | + | + |
| *SNTA1* | + | + | + |
| *SNX7* |  |  | + |
| *SPCS3* | + |  | + |
| *SSR3* | + |  |  |
| *STAF65* | + | + |  |
| *TAP1* | + | + | + |
| *TAPBP* | + | + | + |
| *TEX101* |  |  | + |
| *TIF1* | + |  | + |
| *TKT* | + | + | + |
| *TM4SF9* | + | + | + |
| *TMSB4X* | + |  | + |
| *TNFSF10* | + | + | + |
| *TNFSF13B* | + | + | + |
| *TRADD* | + |  |  |
| *TPM1* |  |  | + |
| *TREM1* | + | + | + |
| *TRPM4* | + |  | + |
| *UCP2* | + | + | + |
| *USP3* |  |  | + |
| *VCAM1* | + | + | + |
| *VEGFC* | + | + | + |
| *VKORC1* | + | + | + |
| *VPS13C* | + | + | + |
| *XBP1* | + |  | + |
| *ZC3HDC1* | + |  |  |
| *ZNF25* | + |  | + |
