# Supplementary material for: Platelets Alter Gene Expression Profile in Human Brain Endothelial Cells in an In Vitro Model of Cerebral Malaria
Source: PLoS One. 2011 May 16;6(5):e19651. doi: 10.1371/journal.pone.0019651 (PMC3095604; doi:10.1371/journal.pone.0019651)
Supplement: Table S3 — List of genes transcriptionally altered by time. (DOC) [file pone.0019651.s004.doc]

**Table S3. List of genes transcriptionally altered by time.**

| Gene | SAM | | t-test | | GeneAnova | |
| --- | --- | --- | --- | --- | --- | --- |
| *ACTA1* | | + | | + | | + |
| *ACTB* | | + | | + | | + |
| *ADAR* | | + | | + | | + |
| *ANTXR2* | | + | |  | | + |
| *ARCN1* | | + | |  | | + |
| *ARHGEF19* | | + | |  | |  |
| *ARL6IP2* | |  | |  | | + |
| *ARMCX1* | | + | | + | | + |
| *ASS* | | + | | + | | + |
| *ATF4* | | + | | + | | + |
| *AURKB* | | + | |  | |  |
| *AVPI1* | | + | | + | | + |
| *BCAT1* | | + | |  | | + |
| *BCL2* | | + | | + | | + |
| *BCL2L13* | | + | |  | | + |
| *BDKRB2* | | + | |  | |  |
| *CAMK1G* | | + | | + | | + |
| *CARD10* | | + | | + | | + |
| *CCL2* | | + | |  | |  |
| *CD74* | | + | | + | | + |
| *CD83* | | + | |  | |  |
| *CDC26* | | + | |  | |  |
| *CDC42EP1* | | + | |  | |  |
| *CDKN3* | | + | |  | |  |
| *CDR2* | | + | | + | | + |
| *CIRBP* | | + | | + | | + |
| *CLK3* | | + | | + | | + |
| *CLN6* | | + | |  | | + |
| *CNGB3* | | + | |  | |  |
| *COL9A2* | | + | | + | | + |
| *CTGF* | | + | |  | | + |
| *DAB2* | | + | |  | | + |
| *DDT* | | + | | + | | + |
| *DNCH1* | | + | |  | |  |
| *DNAJB9* | |  | |  | | + |
| *DUSP10* | | + | | + | | + |
| *DUSP5* | | + | | + | | + |
| *E2F2* | | + | |  | | + |
| *EIF3S8* | | + | | + | | + |
| *ELK3* | | + | | + | | + |
| *ENG* | | + | |  | | + |
| *ETV6* | | + | |  | |  |
| *EVI1* | |  | |  | | + |
| *FAM13A1* | | + | |  | | + |
| *FCHO1* | | + | | + | | + |
| *FOSB* | | + | | + | | + |
| *FOSL2* | | + | |  | |  |
| *GAS1* | | + | | + | | + |
| *GBE1* | | + | |  | |  |
| *GBP5* | | + | | + | | + |
| *GLIS2* | | + | |  | |  |
| *GLYAT* | | + | | + | |  |
| *GPATC1* | | + | |  | |  |
| *H1FX* | | + | | + | | + |
| *HBEGF* | | + | |  | |  |
| *HIF1AN* | | + | |  | | + |
| *HIST2H2AA* | | + | |  | | + |
| *HIST4H4* | | + | | + | | + |
| *HMGB3* | |  | |  | | + |
| *HSF2* | |  | |  | | + |
| *HSPCB* | | + | |  | | + |
| *ICAM1* | | + | | + | | + |
| *ID3* | | + | | + | | + |
| *IFI35* | | + | |  | |  |
| *IFIT3* | | + | | + | | + |
| *IL11* | | + | | + | | + |
| *IL22RA2* | | + | |  | | + |
| *IL2RG* | | + | |  | |  |
| *IMP-2* | | + | |  | |  |
| *IRS1* | | + | |  | | + |
| *KCTD2* | | + | |  | | + |
| *KLF10* | | + | | + | | + |
| *KRT10* | | + | | + | | + |
| *KRT5* | | + | |  | | + |
| *LAP3* | | + | |  | | + |
| *LCAT* | | + | | + | |  |
| *LEPRE1* | |  | |  | | + |
| *LRPAP1* | | + | | + | | + |
| *LRRC8* | | + | | + | | + |
| *MAP1A* | | + | |  | |  |
| *MARK2* | | + | |  | |  |
| *MCEE* | | + | | + | | + |
| *MED18* | | + | | + | | + |
| *MEIS2* | | + | |  | | + |
| *MICAL-L1* | | + | |  | |  |
| *MNDA* | |  | |  | | + |
| *MRPL17* | | + | | + | | + |
| *MX2* | | + | | + | | + |
| *MYD88* | | + | | + | |  |
| *NAV1* | | + | | + | | + |
| *NEUROD6* | | + | | + | | + |
| *NP_079363.1* | | + | | + | | + |
| *NPD014* | | + | | + | | + |
| *NT5C3* | | + | |  | |  |
| *NUDT5* | | + | |  | |  |
| *OAS1* | | + | | + | | + |
| *OGDH* | | + | |  | | + |
| *PAK1IP1* | | + | | + | | + |
| *PDE7B* | | + | | + | | + |
| *PGCP* | | + | | + | | + |
| *PHLDA1* | | + | | + | | + |
| *POP5* | | + | | + | | + |
| *POR* | | + | |  | |  |
| *PPP4R2* | |  | |  | | + |
| *PTPNS1L2* | | + | |  | | + |
| *RAB39B* | | + | | + | | + |
| *RAPGEF1* | | + | |  | |  |
| *raptor* | | + | |  | |  |
| *RASGRP3* | | + | |  | |  |
| *RGS19IP1* | | + | | + | | + |
| *RHOJ* | | + | |  | | + |
| *RIT1* | | + | | + | | + |
| *RPIA* | | + | |  | | + |
| *RPL12* | | + | | + | | + |
| *RPL36* | |  | | + | | + |
| *RPL22L1* | | + | |  | |  |
| *RPLP0* | | + | |  | |  |
| *RRM2B* | | + | |  | |  |
| *RREB1* | |  | |  | | + |
| *SEC11L2* | | + | |  | | + |
| *SELB* | | + | |  | | + |
| *SELM* | | + | | + | | + |
| *SELS* | | + | | + | | + |
| *SFPQ* | | + | |  | |  |
| *SH2D3A* | | + | | + | | + |
| *SLC35A4* | | + | |  | |  |
| *SLC38A1* | | + | | + | | + |
| *SLC38A2* | | + | | + | | + |
| *SLC41A2* | | + | |  | |  |
| *SLC9A3R2* | | + | |  | | + |
| *SLC9A9* | | + | | + | | + |
| *SMC4L1* | | + | | + | | + |
| *ST5* | | + | |  | |  |
| *STARD4* | | + | |  | |  |
| *STARD5* | | + | |  | | + |
| *SUI1* | | + | |  | |  |
| *TCEAL3* | | + | |  | |  |
| *TCTEL1* | | + | |  | |  |
| *TIGA1* | | + | | + | | + |
| *TIMP1* | | + | | + | | + |
| *TLR4* | | + | | + | | + |
| *TOB1* | | + | |  | | + |
| *TPD52L1* | | + | |  | | + |
| *TRAF2* | | + | | + | | + |
| *TRAF6* | | + | |  | |  |
| *TRIP6* | | + | |  | | + |
| *TRUB2* | | + | |  | | + |
| *Tst* | | + | |  | |  |
| *TTN* | |  | | + | | + |
| *Usp18* | | + | | + | | + |
| *USP25* | | + | |  | |  |
| *VPS18* | | + | |  | | + |
| *WRB* | | + | | + | |  |
| *XPO1* | | + | |  | |  |
| *XRCC3* | | + | |  | | + |
| *XTP3TPA* | | + | |  | |  |
| *ZNF133* | | + | | + | | + |
| *ZNRF2* | | + | |  | |  |
