# Supplementary material for: Platelets Alter Gene Expression Profile in Human Brain Endothelial Cells in an In Vitro Model of Cerebral Malaria
Source: PLoS One. 2011 May 16;6(5):e19651. doi: 10.1371/journal.pone.0019651 (PMC3095604; doi:10.1371/journal.pone.0019651)
Supplement: Table S4 — IPA Network analysis based on the 58 platelet-regulated genes. (DOC) [file pone.0019651.s005.doc]

**Table S4. IPA Network analysis based on the 58 platelet-regulated genes.** We selected networks with a Score>3 (*P*<0.001). Molecules in bold-faced type highlight platelet-regulated genes in our study that are included in these networks.

| Network | Molecules | Top Functions | Score |
| --- | --- | --- | --- |
| 1 | Ap1,**BCL3**,**CCL2**^a^,**CCL4**,**CCL7**,**CCR7**,**CD83**,**CXCL1**,**CXCL3**,**DUSP5**^a^,ERK,**FCER1G**,**FOSB**,**ID3,IER3**^a^,IFNBeta,IKK,IL1,**IL11**,IL12p70,**INHBA**,Interferon alpha,**IRF1**,LDL,**MSC**,**MUC4**,NfkB-RelA,P38MAPK,Pdgf,PDGFBB,**PLK2**,STAT5a/b,Tgf beta,**TNNI3** | Cellular Movement, Hematological System Development and Function, Immune Response | 44 |
| 2 | ADAM17,**ADAM21**,**ADAM28**,AGT,APH1A,**APH1B**,BDKRB2,CDH1,CDKN1B,COPS3,COPS8,COPS7A,**COPS7B**,**DDIT4**,GNRHR,**H1FX**,HSD3B1,**IER3**^a^,IGF1,**KCNK3**,LRP1B,Metalloprotease,Pde,**PDE1A**,**PDE7B**,PDE8A,Pkc(s),PLA2G6,**PRG2**,**RNF144B**,SH3BGRL2,**SORL1**,TACSTD1,TP53 | Cancer, Gastrointestinal Disease, Cellular Growth and Proliferation | 26 |
| 3 | beta-estradiol,CLDN4,**DNASE1L3**,**DUSP5**^a^,**EHD1**,**EPHA2**,FYN,GNL3,HAS1,**IER3**^a^,IGSF1,IL2,IL18BP,IL1B,IL1F6,**INHBB**,KCND3,**KCNIP3**,KRT6A,MGP,NCR1,NRTN,OAS2,PGDS,PI3,**RAVER1**,**RCAN3**,**SACS**,TNF,TNFAIP8^a^,TNFSF11,TPD52L2,**UAP1**,**UCK2** | Cardiovascular Disease, Cellular Compromise, Cell Signaling | 23 |
| 4 | Akt,**BIRC2**,Caspase,Ck2,**CTGF**,CXCL16,EDA2R,**GLB1**,**HBEGF**,Hsp70,Insulin,Jnk,Mapk,**NDUFA1**,NFkB,NfkB-Nfkbia,**NFKBIA**,NIBP,NKIRAS1,**ODC1**^a^,Ornithine decarboxylase,**PAK1IP1**,PI3K,PIF,Pka,PLC,PRDX4,Proteasome,**PTK6**,SUMO4,TNAP,TNFAIP8^a^,TNIP3,**TPD52L1**,**TRAF4** | Metabolic Disease, Renal and Urological Disease, Lipid Metabolism | 21 |
| 5 | ACVR1,aminoacids,**ANGPTL4**,ATM,BYSL,CASP7,**CCL2**^a^,CCL11,CITED2,FRAP1,IGFBP3,IL17A,JAK1,KRT8,KRT18,LPL,MAP2K1,MAP2K6,MEF2C,**ODC1**^a^,p70,PAK1,PAK3,PDPK1,**PGCP**,RPS6KA1,RPS6KB1,SGK1,**SRPK3**,TGFB1,TGFBR2,TRO,**TROAP**,TYK2,YWHAE | Amino Acid Metabolism, Post-Translational Modification, Small Molecule Biochemistry | 10 |

^a^ Genes shared by two or more networks.
