# Supplementary material for: Platelets Alter Gene Expression Profile in Human Brain Endothelial Cells in an In Vitro Model of Cerebral Malaria
Source: PLoS One. 2011 May 16;6(5):e19651. doi: 10.1371/journal.pone.0019651 (PMC3095604; doi:10.1371/journal.pone.0019651)
Supplement: Table S5 — IPA Canonical Pathways analysis based on the 58 platelet-regulated genes. (DOC) [file pone.0019651.s006.doc]

**Table S5. IPA Canonical Pathways analysis based on the 58 platelet-regulated genes.**

| Canonical Pathway name | *P*-value |
| --- | --- |
| Hepatic Fibrosis | 2.10^-3^ |
| Death Receptor Signaling | 2.10^-3^ |
| Aminosugars Metabolism | 3.10^-3^ |
| Chemokine Signaling | 4.10^-3^ |
| Apoptosis Signaling | 6.10^-3^ |
| G-Protein Coupled Receptor Signaling | 9.10^-3^ |
| Glucocorticoid Receptor Signaling | 2.10^-2^ |
| IL-10 Signaling | 3.10^-2^ |
| Hypoxia Signaling in the Cardiovascular System | 3.10^-2^ |
| Erythropoietin Signaling | 3.10^-2^ |
| LXR/RXR Activation | 4.10^-2^ |
| TGFβ Signaling | 4.10^-2^ |
